# From Drug-Induced Developmental Neuroapoptosis to Pediatric Anesthetic Neurotoxicity—Where Are We Now?

**DOI:** 10.3390/brainsci6030032

**Published:** 2016-08-16

**Authors:** Catherine E. Creeley

**Affiliations:** Department of Psychology, State University of New York at Fredonia, Fredonia, NY 14063, USA; creeley@fredonia.edu; Tel.: +1-716-673-3890

**Keywords:** early brain development, drugs, cognitive development, neurodevelopment

## Abstract

The fetal and neonatal periods are critical and sensitive periods for neurodevelopment, and involve rapid brain growth in addition to natural programmed cell death (i.e., apoptosis) and synaptic pruning. Apoptosis is an important process for neurodevelopment, preventing redundant, faulty, or unused neurons from cluttering the developing brain. However, animal studies have shown massive neuronal cell death by apoptosis can also be caused by exposure to several classes of drugs, namely gamma-aminobutyric acid (GABA) agonists and *N*-methyl-d-aspartate (NMDA) antagonists that are commonly used in pediatric anesthesia. This form of neurotoxic insult could cause a major disruption in brain development with the potential to permanently shape behavior and cognitive ability. Evidence does suggest that psychoactive drugs alter neurodevelopment and synaptic plasticity in the animal brain, which, in the human brain, may translate to permanent neurodevelopmental changes associated with long-term intellectual disability. This paper reviews the seminal animal research on drug-induced developmental apoptosis and the subsequent clinical studies that have been conducted thus far. In humans, there is growing evidence that suggests anesthetics have the potential to harm the developing brain, but the long-term outcome is not definitive and causality has not been determined. The consensus is that there is more work to be done using both animal models and human clinical studies.

## 1. Introduction

Apoptosis, known as one form of programmed cell death (PCD) or cell suicide, is a natural process by which neurons in the developing brain are selectively deleted in a process that contributes to normal neurodevelopment, before or after neuronal differentiation. PCD may delete 50% or more of all newly formed neurons, an important process critical for the formation of a highly structured and efficient fully formed human brain. In the periphery, PCD was revealed to be regulated by the competition for a limited amount of nerve growth factor (NGF), which allows for a pattern of connections to form between proliferating and target cells [1,2,3]. In the central nervous system (CNS), however, the process is more complex, relying on neuronal activation and neurotransmitter activity rather than the simple presence of growth factors [4,5,6]. Abnormal patterns of apoptosis can be triggered by altering neurotransmitter activity through exposure to psychoactive drugs. The amount of drug-induced apoptotic cell death depends on timing, dose, and duration, and can be a significant adverse event that results in a widespread neurodegeneration throughout the developing brain, which is particularly vulnerable to environmental insult during the fetal and neonatal period [7]. Of particular concern is the effect that drugs have on the developing brain, as exposure to a wide variety of drugs may significantly alter the course of neurodevelopment, which may lead to permanent changes and a lifetime of intellectual disability that includes both cognitive and behavioral deficits [8,9,10,11,12]. An underlying factor in the alteration of brain development may be drug-induced neurodevelopmental apoptosis (DIDNA). DIDNA was first identified in the neonatal rodent brain, caused by classes of drugs that have *N*-methyl-d-aspartate (NMDA) receptor antagonist or gamma-aminobutyric acid (GABA) mimetic properties, such as ethanol (which has both properties) [13,14,15,16,17], some drugs of abuse [18,19,20], anticonvulsants [21,22], sedatives, and anesthetics [23,24,25,26,27,28]. The results of early studies provided evidence that pointed to the fact that this type of neurotoxicity is a major public health concern if agents administered in obstetric and pediatric medicine could have the potential to cause brain damage. It is also not uncommon for pregnant women to abuse drugs, or to undergo therapeutic treatment that involves the use of anticonvulsants, sedatives, and anesthetics [8]. The premature infant is also routinely exposed to sedatives, analgesics, and anesthetics, sometimes for prolonged periods during a stay in the neonatal intensive care unit. Neonates and infants also may be exposed to sedatives and anesthetics for procedural sedation and surgery [29].

Results of years of research have shown that, in animals, DIDNA may cause permanent changes in brain receptor function and circuitry that results in sensorimotor, social, and cognitive deficits [30]. In humans, fetal or neonatal drug exposure has been linked to learning disabilities and behavioral/developmental deficits [8]. This converging evidence led researchers to propose the need to investigate the potential neurotoxicity of pediatric sedatives and anesthetics [31]. The objective of this paper is to review the seminal animal experiments on DIDNA and examine the current status of the evidence regarding pediatric anesthetic neurotoxicity in humans.

## 2. Methods

This is a review of the research that has been a part of a major scientific movement in the field to design and conduct animal and human studies that set out to answer the research question as to whether or not pediatric sedatives and anesthetics are neurotoxic to the developing brain. This review begins with the seminal research from the laboratory of John W. Olney beginning in 1999 [25], and follows the progress from there, ending with the most recent clinical studies that have been completed as of June 2016. The PubMed database was used to search for relevant literature using keyword searches for original research and review articles that included the following terms: anesthesia/anesthetics, pediatric anesthesia/sedation, neurodevelopment, apoptosis, and neurodegeneration.

## 3. DIDNA

The finding that drug exposure can cause changes in the neurodevelopment is decades old, with an early focus mostly on drugs of abuse such as alcohol [32] and morphine [33]. There were few studies on therapeutic exposure to sedatives and anesthetics [23,24]. The discovery of DIDNA was first identified by the Olney lab at Washington University School of Medicine in St. Louis when Ikonomidou et al. [25] found that NMDA antagonist drugs caused widespread apoptotic cell death in the neonatal rat brain. In a series of subsequent reports from the Olney lab they showed that a similar effect could be observed in the rat or mouse brain by several other types of drugs, including γ-aminobutyric acid type A (GABA_A_) mimetics, and drugs with combined GABA mimetic and NMDA antagonist properties, such as ethanol, anticonvulsants, drug combinations, and anesthetic agents such as ketamine, midazolam, propofol, and isoflurane [13,14,15,20,25,26,27,28]. It was determined, and is now widely accepted, that the brain growth spurt period is when the fetal or infant brain is most vulnerable to DIDNA. In rodents this period is postnatal, during the first week of life. In the human brain, this period begins during fetal development and extends throughout early childhood [34]. Vulnerability to DIDNA is not limited to just one brain region, and has been found to occur through the entire brain including the forebrain, midbrain, cerebellum, and visual system [13,14,15,35,36]. The pattern of cell death is dependent on the timing of exposure during synaptogenesis. The underlying mechanism was shown to be Bax-dependent and to involve mitochondrial injury, extramitochondrial leakage of cytochrome c, and activation of caspase-3 [37,38,39]. Subsequent studies provided evidence that brain-derived neurotrophic factor-dependent and death receptor-dependent pathways were also involved [39,40,41].

This early evidence was not immediately accepted—the translational relevance to the human clinical experience was questioned, based on observations that the doses and durations used in the animal models were too high and for too long, respectively [42,43,44]. It was subsequently argued that a clinically relevant dose is dependent upon effect for the given species. For example, the doses of anesthetics that should be tested are those that produce a specified anesthetic effect in animals or humans, and these doses may not be the same on a strictly mg/kg basis. When this metric based on a clinically effective dose is applied, the cross-species comparison can be made [45]. In the next 10 years, DIDNA was confirmed by multiple labs using in vitro and in vivo animal models, including the non-human primate [46,47,48,49,50]. As the debate among researchers and clinicians began about whether these results should influence clinical practice [51,52,53], multiple reviews of the animal research all came to the same conclusion—results animal studies on drug-induced neurotoxicity were robust enough to suggest that anesthetics and sedatives used in pediatric and obstetric medicine may have the ability to damage the developing brain, and clinical research was needed [54,55,56,57,58]. The research focus moved from the preclinical to the clinical realm, leading to the establishment of national and international research consortiums, groups, and organizations devoted to determining if anesthetics pose a significant risk to the developing human brain [59,60,61,62].

## 4. Clinical Investigations

Certainly the discovery and documentation of fetal alcohol syndrome as early as the 1970s was an early indicator that there could be serious consequences to exposing the developing brain to other psychoactive drugs [32], especially those that, either alone or in combination, influence GABA agonist and NMDA antagonist activity in the brain. Based on the initial results published by Olney and colleagues [13,14,15,25,26,27,28], pediatric anesthetic regimens were identified as suspect because they use drugs that have GABA mimetic or NMDA antagonist properties. Researchers began clinical retrospective research using archived data sets from a population of children, already exposed to anesthesia, available for analysis. Respectably, an international clinical research community set to work. In 2009, published results of retrospective studies of children exposed to anesthesia before age 3 suggested that there may be an association between early exposure to anesthesia and an increased risk of behavioral/developmental disorders [63,64,65]. The results of these studies were consistent with earlier studies that, until this point, had not received much attention from clinicians and researchers in the field [66,67]. Taken together, this evidence suggested that early anesthesia exposure could result in poor neurodevelopmental outcomes that persist into childhood.

Not all studies found an association, however [68], and this early research did not immediately sway clinical opinion, as prospective studies had not been conducted and causality could absolutely not be determined [69,70,71,72]. Subsequent research added to the evidence that neonatal exposure to anesthesia may, or may not, have long-term adverse effects on behavioral and cognitive development (see Table 1). Overall, the findings suggest that early multiple exposures to surgery/anesthetics may increase the risk for a behavioral developmental disorder, but a single exposure likely does not [73,74,75,76].

In an attempt to synthesize the clinical data from retrospective studies, DiMaggio et al. [87] conducted a search of studies published from 2002 to 2012 [63,64,65,68,73,74,75,77,78,79,80,88] and conducted a Bayesian analysis of selected studies [63,65,68,77,78,80,88]. The authors concluded that the results indicated a modest, but elevated risk of adverse behavioral/developmental outcomes, but that the uncertainty of the available epidemiological evidence might preclude further research using existing data sources. The focus then shifted to studies designed to investigate associations between anesthetic exposure and a diagnosis for specific disorders such as learning disorder (LD), attention-deficit/hyperactivity disorder (ADHD), and autism spectrum disorders (ASD), but no associations were found [66,67,82,83,84]. It has since been suggested that studies should be designed that focus on more specific outcome measures, because the variability in the results of clinical data may be due to lack of consistent and well-defined dependent measures, such as tests designed to assess specific cognitive and behavioral deficits, rather than more global measures using an intelligence quotient (IQ) or performance on academic achievement tests. For example, Ing et al. [81] did show that results were dependent on clinical outcome measures used—there was evidence for an increased risk for specific cognitive deficits, but no increased risk was observed when academic achievement results were analyzed. This new focus on outcomes could lead to future studies that reveal deficits to be domain-specific and may be unable to be discovered using global measures such as IQ, LD diagnoses, or group academic achievement tests. Future research is dedicated to minimizing confounds, maximizing control, and using specific test batteries to characterize a neurobehavioral phenotype that is specifically associated with anesthetic neurotoxicity [81,84,85,86,89,90,91,92]. New patient populations are also being considered [93,94], but are complicated and have raised important new questions about potential perioperative risks that are also potential sources of brain injury (i.e., hypotension, hypoxia/ischemia, hypocapnia) [95]. The debate among researchers and clinicians has been vigorous and productive—several review articles have been written in the past few years that simultaneously acknowledge a potential risk and conclude that we simply cannot know if anesthesia causes neurotoxic effects in the human brain that would require a change in clinical practice or if this risk should be included as part of informed consent. Most agree, however, that important questions remain that cannot be answered without continued preclinical and clinical research [95,96,97,98,99,100,101,102,103].

## 5. Conclusions

### Summary of Findings

- There is abundant evidence from animal research that suggests that pediatric sedation and anesthesia has the potential to be neurotoxic in the developing brain.
- Studies in humans are mostly retrospective, and have found that a single exposure to anesthesia is likely not a concern, but it cannot be ruled out that multiple exposures could increase the risk for poor neurodevelopmental outcome.
- Prospective human clinical research is needed; retrospective studies should focus on neurobehavioral and domain-specific outcome measures rather than diagnoses or global outcomes such as standardized test and academic achievement scores.
- New research in animals should focus on finding the safest protocols, drugs, and drug combinations.
- There is not enough evidence to prompt significant changes in clinical practice, but elective or non-urgent surgeries should be avoided.
- More research is needed – preclinical and clinical.

There is no question that this issue has become a global health concern, and a network of research labs and groups has been actively pursuing answers that will lead to best practice. It is also the case that the issue of anesthetic neurotoxicity is debatable—not everyone is convinced that it is anesthesia that presents the greatest risk. Inherent limitations to research with human participants that start with the intrinsic differences within the patient population, and are often underpowered and heavily confounded by differences among and between groups place major limitations on our ability to accurately link anesthesia to long-term developmental outcomes. It should also be noted that other drugs that do not cause apoptotic neurodegeneration in the brain are also linked to poor neurodevelopmental outcomes. The simple fact of the matter is that exposure to anesthesia is unavoidable and necessary to mitigate pain and distress, which itself has been shown to have adverse effects on development. Future directions are to conduct more prospective studies and to design more controlled retrospective studies that include the use of domain-specific outcome measures. The overall goal is to prevent or limit any potential brain injury using evidence-based clinical strategies. The evidence thus far is not strong enough to change clinical practice, but has asked and answered important questions that have rightly advanced our knowledge in significant ways.

## Figures and Tables

**Table 1:** Clinical research conducted to investigate the effects of early exposure to anesthesia, in order by publication date.

First Author	Date	Age at Exposure	Study Groups	Drugs	Study Design and Population	Outcome Measure and Endpoint	Conclusions
Roze [77]	2008	<33 weeks	Exposed (*n* = 115); non-exposed (*n* = 1457)	Daily exposure to sedatives and/or opioids	Prospective, population-based study, France	Presence of disability at age 5	Prolonged sedation/analgesia (>7 days) not associated with poor outcome
Kalkman [78]	2009	<2 years	<24 months (*n* = 178); >24 months (*n* = 65)	Isoflurane, halothane, enflurane, fentanyl, sufentanil	Retrospective cohort study, Netherlands	Child Behavior Checklist	No ability to confirm an effect, study underpowered
Wilder [65]	2009	<4 years	No exposure (*n* = 4,764); Single (*n* = 449); Multiple (*n* = 144)	Isoflurane, halothane, enflurane, sodium thiopental, etomidate, ketamine, nitrous oxide, diazepam	Population-based, retrospective birth cohort study; USA	Reading, language, and math LDs before age 19	Significant increased risk of LD with multiple, but not single exposure
Bartels [68]	2009	<3 years	Exposure before age 3 vs. exposure age 3–12; *n* = 1143 twin pairs	Information not available to researchers	Monozygotic concordant-discordant twin study, Netherlands Twin Registry	Educational achievement and cognitive problems at age 12	No difference between exposed and unexposed twin
Sprung [64]	2009	Perinatal	Cesarian (*n* = 497) vs. vaginal delivery (*n* = 4823)	Isoflurane, halothane, enflurane, sodium thiopental, etomidate, ketamine, nitrous oxide, methoxyflurane	Population-based birth cohort: USA	Incidence of learning disability (LD) before age 19	No association between anesthetic exposure during birth and risk of LD
DiMaggio [63]	2009	≤3 years	Hernia repair (*n* = 383) vs. Age-matched controls (*n* = 5050)	Not provided	Retrospective cohort study of hernia patients; NY State Medicaid Program enrollees, USA	Risk of behavioral/develop-mental disorder diagnosis at or before age 4	Children who had hernia repair >2x as likely to be diagnosed
Hansen [79]	2011	<1 year	Hernia repair (*n* = 2689) vs. Age-matched controls (14,575)	Not provided	Retrospective birth cohort study, Denmark	Academic achievement test at 15 or 16 years	No evidence of any effects of a single exposure
Guerra [80]	2011	≤6 weeks	Cardiac surgery (*n* = 95) dose-response	Inhalationals, opioids, benzodiazepines, ketamine, chloral hydrate	Prospective post-operative follow-up	Mental, motor, and vocabulary assessment at 18–24 months	No association between dose/durationof sedation/analgesia and neurodevelopmental outcome
DiMaggio [73]	2011	<3 years	Surgery before age 3 (1–3 exposures) (*n* = 304) vs. unexposed controls (*n* = 10,146)	Information not available to researchers	Retrospective sibling birth cohort design; NY State Medicaid Program enrollees, USA	Incidence of developmental and behavioral disorder at or before age 6	Anesthesia-exposed group risk of diagnosis 60% higher; no causal connection can be made
Flick [74]	2011	<2 years	General anesthesia exposure once (*n* = 286), more than once (*n* = 64) vs. unexposed controls (*n* = 700)	Combination of halothane and nitrous oxide (most common)	Retrospective matched cohort study, USA	LD diagnosis, achievement, cognitive tests before age 19	Multiple exposures to anesthesia increases risk for a LD, but no intervention required
Sprung [75]	2012	<2 years	No exposure (*n* = 10,146); single exposure (*n* = 210); two exposures (*n* = 71); three or more (*n* = 23)	Combination of halothane and nitrous oxide (most common)	Retrospective birth cohort design, USA	Attention-deficit/hyperactivty (ADHD) diagnosis before age 19	No increased risk with single exposure, but increased risk for ADHD diagnosis with repeated exposure
Ing [81]	2014	<3 years	Disability class: none (*n* = 1135); language and cognitive (*n* = 96); behavioral deficits (*n* = 151); severe deficits (*n* = 62)	Information not available	Retrospective birth cohort study, Western Australian Pregnancy Cohort (Raine) Study	Language, cognition, motor skills and behavior at age 10	Deficits in language and abstract reasoning associated with anesthesia exposure. Phenotype of interest may be specific language/cognitive delays
Ko [82]	2014	<3 years	Exposed (*n* = 3293) vs. unexposed (*n* = 16,465)	Sevoflurane	Population-based retrospective matched birth cohort design, National Health Insurance Database of Taiwan	Risk of ADHD before age 10	No increased risk of ADHD diagnosis for single or multiple exposure
Ko [83]	2015	<2 years	Exposed (*n* = 5197) vs. unexposed (*n* = 20,788)	Sevoflurane	Population-based, retrospective matched birth cohort design, National Health Insurance Database of Taiwan	Risk of autism disorder diagnosis before age 10	No increased risk of AD; no relationship between total number of exposures and AD risk
Creagh [84]	2015	<3 years	ASD diagnosed (*n* = 262) vs. non-ASD (*n* = 253)	In utero exposure—specific agents not known	Population-based sibling cohort study, Puerto Rico	Risk of autism spectrum disorder (ASD) diagnosis	Early exposure to anesthesia not linked to an ASD diagnosis
Gleich [85]	2015	<3 years	No exposure (*n* = 250); single (*n* = 150 ); multiple (*n* = 100); Hypothetical sample sizes	Data to be collected	Population-based, retrospective propensity-matched study, USA	Neuropsychological test battery (also used in primates), ages 8–12 or 15–19	Analyses not completed; goal is to determine whether a neurobehavioral phenotype exists
Hu [86]	2016	<3 years	No exposure (*n* = 465); single exposure (*n* = 466); multiple exposure (*n* = 126)	Data to be collected	Population-based, retrospective birth cohort	LD or ADHD diagnosis, group achievement test at age 5 or 6	Only slight differences between study groups, not expected to affect future data analysis comparing risk
Sun [76]	2016	<3 years	Sibling pairs (*n* = 105); single exposure vs. unexposed	Inhaled anesthetic agents	Sibling matched cohort study, at 4 U.S. University-based hospitals	Global cognitive function (IQ) at age 10	No risk for healthy children with single exposure
